# Role of Phage Capsid in the Resistance to UV-C Radiations

**DOI:** 10.3390/ijms22073408

**Published:** 2021-03-26

**Authors:** Laura Maria De Plano, Domenico Franco, Maria Giovanna Rizzo, Vincenzo Zammuto, Concetta Gugliandolo, Letteria Silipigni, Lorenzo Torrisi, Salvatore P. P. Guglielmino

**Affiliations:** 1 Department of Chemical, Biological, Pharmaceutical and Environmental Sciences, University of Messina, 98166 Messina, Italy; ldeplano@unime.it (L.M.D.P.); mgrizzo@unime.it (M.G.R.); vzammuto@unime.it (V.Z.); cgugliandolo@unime.it (C.G.); sguglielm@unime.it (S.P.P.G.); 2 Department of Mathematical and Computational Sciences, Physical Sciences and Earth Sciences, University of Messina, 98166 Messina, Italy; lsilipigni@unime.it (L.S.); ltorrisi@unime.it (L.T.)

**Keywords:** M13 engineered phage, UV-C, ionizing direct and indirect damage, virus stability

## Abstract

The conformational variation of the viral capsid structure plays an essential role both for the environmental resistance and acid nuclear release during cellular infection. The aim of this study was to evaluate how capsid rearrangement in engineered phages of M13 protects viral DNA and peptide bonds from damage induced by UV-C radiation. From in silico 3D modelling analysis, two M13 engineered phage clones, namely P9b and 12III1, were chosen for (i) chemical features of amino acids sequences, (ii) rearrangements in the secondary structure of their pVIII proteins and (iii) in turn the interactions involved in phage capsid. Then, their resistance to UV-C radiation and hydrogen peroxide (H_2_O_2_) was compared to M13 wild-type vector (pC89) without peptide insert. Results showed that both the phage clones acquired an advantage against direct radiation damage, due to a reorganization of interactions in the capsid for an increase of H-bond and steric interactions. However, only P9b had an increase in resistance against H_2_O_2_. These results could help to understand the molecular mechanisms involved in the stability of new virus variants, also providing quick and necessary information to develop effective protocols in the virus inactivation for human activities, such as safety foods and animal-derived materials.

## 1. Introduction

Viruses are biological entities, consisting of a single element of nucleic acid (RNA or DNA, in either single- or double-stranded forms) enclosed in a protective protein shell, or capsid, and are only able to replicate inside living cells. The virus’s resistance to environmental stresses (temperature, pH, interaction with host cells, etc.) is closely related to the protein composition of the external structures of the virion. This is generally made up of repeated protein sub-structures, named capsomers, organized in polyhedral or helical symmetry [1]. These simple geometries can be formed with a limited number of capsid protein subunits, increasing their volume easily by repeatedly using the same proteins and consequently limiting the number of genes required for the synthesis of the capsid. The arrangement of capsid proteins in helical symmetry is a highly effective way to form tridimensional structures with repetitive subunits that, following the symmetry of the nucleic acid, have the same identical interactions that capsomeres have with each other. Therefore, modifications of capsid proteins, even of a single amino acid, may change the stability and/or metastability of the virion particles [2]. This eventuality is critical for the risk of viral infection, only reduced by several control techniques, such as heat sterilization, chemical disinfectants, air filtration, and ultraviolet (UV) irradiation. In particular, disinfection using UV radiation is a fast-growing because it is chemical-free technology and with low risk of damage to materials (especially heat-labile ones) [3]. For this reason, main parameters should be considered for the elaboration of protocols to guarantee the efficacy of the UV sterilization process, mainly in the latest period strongly marked by COVID-19 pandemic, where UV disinfection of surfaces and air has attracted tremendous attention [4].

Bacteriophages (or simply phage) are viruses that infect bacteria and have several biotechnological applications, including their use as models for human viruses. Specifically, M13 phage is well known for the development of the phage display technique, which allows at foreign peptides to be exposed on the virion capsid [5]. The capsid of M13 phage is characterized by a helical symmetry structure, almost exclusively constituted by the major coat protein pVIII (responsible for phage integrity), and four other minor coat proteins, namely pIII, pV, pVII and pIX, which together enclose the circular single-stranded DNA molecule [6]. In the phage display application, the engineering with foreign peptides in the pVIII protein (representing about 98% of capsid proteins) permits the formation of landscape phage libraries of billions of clones, wherein each individual phage displays a random peptide on its surface. Phage libraries based on vector systems (88 or 8 + 8) result in a hybrid structure with mixture of recombinant and wild-type pVIII molecules on the phage capsid [7]. These random phage libraries are used in screening processes against desired targets to isolate specific ligands in the marker discovery, in vitro or in vivo [8,9,10], or to functionalize surfaces for biosensors [11,12,13,14], as specific scaffolds for tissue-regenerating [15], for immunization [16], for drug-targeting [17,18,19], for hybrid-materials [20,21,22], for quantum dots, and for semi-conducting or magnetic nanowire devices [23,24]. It is known that the capsid structure has been suggested to be responsible for the great resistance of M13 to various physical and chemical stresses (i.e., heat, both acidic or alkaline organic solvents, and variations in pH values) [25,26]. About the susceptibility to the UV-C exposure, genome type and organization can play an important role against UV inactivation of phage. Phages with circular ssDNA, such as PhiX174, are most sensitive respect to that with linear ssRNA, such as MS2; while phage with linear dsDNA, such as PRD1, revealed the highest photoreactivation after UV exposure [27,28]. In contrast to UV inactivation, MS2 showed a greater susceptibility to gamma or electron beam irradiation compared to PhiX174, whose inactivation revealed to have a clear dose rate effect [29]. Since phages have a limited ability to repair DNA, the shielding of their genomes by capsids may play a key role in their resistance to radiation. Studies on phage T7 would seem to highlight a greater sensitivity towards UV inactivation of intraphage DNA compared to the isolated one, indicating the role of phage proteins in the DNA damage [30]. Moreover, it has been seen that fd virus (with single-stranded nucleic acid) shows a UV sensibility midway between characteristic for the single-stranded and double-stranded nucleic acid viruses [31]. These findings had been attributed to the capsid structure, consisting in a helical structure, with a 60 A pitch and six repeating units per turn that protect the phage nucleic acid from tertiary structural changes induced by the UV-C irradiation [31,32]. On the other hand, effects on biological components from UV-C exposure also involve protein, other than nucleic acids (RNA or DNA) [3]. Although many studies have been focused on the influence of nucleic acid on the UV-C radiation response, at our knowledge, no study has been reported on the role of physical shape and protein on the virus resistance to UV-C or other qualities and quantities of radiations.

The structural model of the M13 capsid, analogue of pC89 wild-type vector, has been used to derive the newly rearrangement and electrostatic interactions due to the addition or modification of amino acids in the capsid structure [6]. In this way, Passaretti et al. investigated the surface charge and the surface area of M13 to predict the mechanisms behind of interactions in bio-nanocomponents, finding that the presence of ionizable groups in foreign peptides can drastically change the chemical characteristics of the engineered virus compared to wild-type, playing a role in the resistance to several stresses [33].

The aim of this study was to evaluate how the capsid rearrangement protects DNA and peptide bonds from damage after exposure to UV-C radiations. By speculative in silico 3D models, two engineered phages, previously selected against *Pseudomonas aeruginosa* (P9b) and antibodies binding β-amyloid structures (12III1), have been chosen for (i) chemical features of amino acids sequences, (ii) rearrangements in the secondary structure of their pVIII proteins and (iii) in turn the interactions involved in phage capsid. Then, their resistances to UV-C radiations were investigated and compared with M13 wild-type vector (pC89) without peptide insert. In addition, the resistance to hydrogen peroxide (H_2_O_2_), one of the most common products generated by the radiolysis of water, has been also evaluated for indirect damage due to the radiation exposure.

## 2. Results

### 2.1. In Silico 3D Models

**Figure 1:**
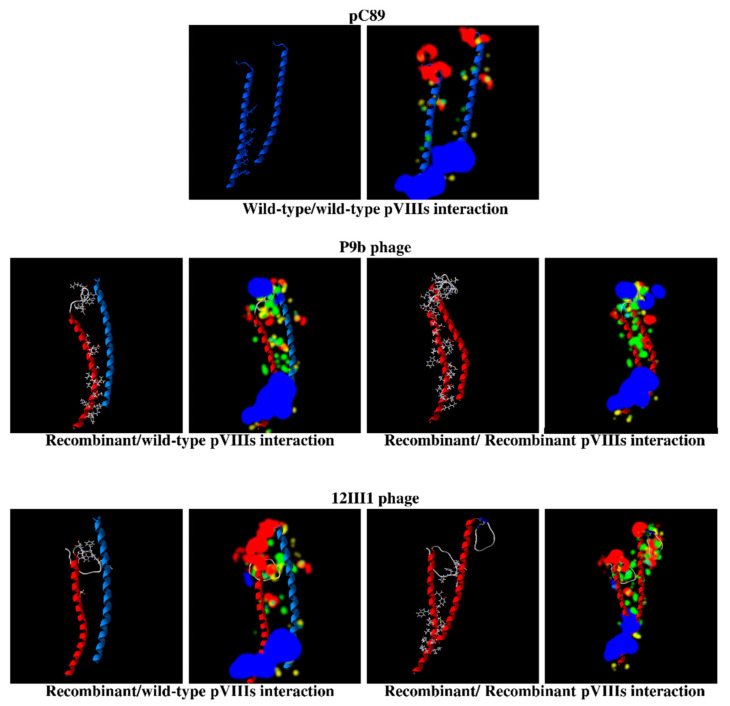
Speculative in silico 3D models of interactions between wild-type (chain blue) and recombinant (chain red) pVIIIs in pC89 (upper panel), P9b (central panel) and 12III1 (lower panel). For each phage, the two pVIII proteins are displayed in the same workspace in backbones style, with the amino-acids of the chains involved in the interaction in wireframe style and coupled to energy map-colored clouds. For energy map, legend colors: in green, the amino acids with stearic favorable bond; in red, a nearby negative electro-static charge of amino acids; in blue, a nearby positive charge of amino acids; in yellow, hydrogens donator favorable; in light blue, hydrogens acceptor favorable.

Engineered phage shows a mosaic of wild-type and recombinant pVIIIs proteins on the capsid structure. The 3D in-silico models provided the constructions of recombinant pVIII proteins with in-frame the amino acids of foreign peptides by Modeller. From 3D models of pVIII proteins, we deduced that the presence of the foreign peptide induced a linear extension of the N-terminal end or a circular form in the same position. In Figure S1 is showed the rearrangement of the secondary structure of the recombinant PVIII of P9b (amino acid sequence of the foreign peptide QRKLAAKLT) and 12III1 (RWPPHFEWHFDD). The interactions between the PVIIIs of the hybrid phage structure, consisting in a mixture of recombinant and wild-type pVIII molecules, also resulted in a new supramolecular rearrangement with new H-bond and steric interactions (Figure 1).

The electrostatic charges highlighted by the colored clouds around the models indicate that the aggregation levels of the chains were modified in all the sequence in P9b and 12III1 phages respect to pC89. 3D in-silico models also allow to derive the amino-acids, including their position number in the primary sequence, which participate in H-bond and steric interactions between the PVIII proteins (in Table 1 and Figure S2).

pC89 showed the lowest number of amino acids involved in the interaction between wild-type pVIIIs (1 and 6 for H-bond and steric interaction, respectively). Otherwise, in P9b phage, amino acids involved in recombinant/wild-type pVIIIs interaction are 5 (H-bond) and 25 (steric interaction), while recombinant/recombinant pVIIIs interaction are 15 (H-bond) and 39 (steric interaction). Finally, in 12III1 phage, amino acids involved in recombinant/wild-type pVIIIs interaction are 1 (H-bond) and 7 (steric interaction), while recombinant/recombinant pVIIIs interaction are 14 (h-bond) and 23 (steric interaction).

### 2.2. UV-C Resistance Test

To assess whether the capsid rearrangement, due to the foreign peptide in PVIII protein, could preserve the infectivity of engineered phages (P9b and 12III1) from effects of radiations, phages were subjected to different UV-C radiations. Before comparative tests between phages, the radio-resistance of pC89 alone was tested up to the lethal dose able to completely reduce the infecting phage load. The last was quantified as Transducing Unit per milliliter (TU/mL), that means number of active virus particles able to infect the bacterial host.

A reduction of about eight orders of magnitude was already found after 1540 J/m^2^, corresponding to 70 s of radiation exposure, while no TU were observed after 3080 J/m^2^ (that means 140 s of radiation exposure).

Based on these findings, the radio-resistances of the engineered phage, P9b (amino acid sequence QRKLAAKLT) and 12III1 (RWPPHFEWHFDD) were compared to that of pC89 up to 3080 J/m^2^ (Table 2).

After UV-C exposure to a 1540 J/m^2^ of fluence the 12III1 and P9b infectivity decreased by about six and four orders of magnitude, respectively. Both engineered phages’ resistance further decreased by about one order of magnitude after the exposure to a 3080 J/m^2^ fluence.

**Figure 2:**
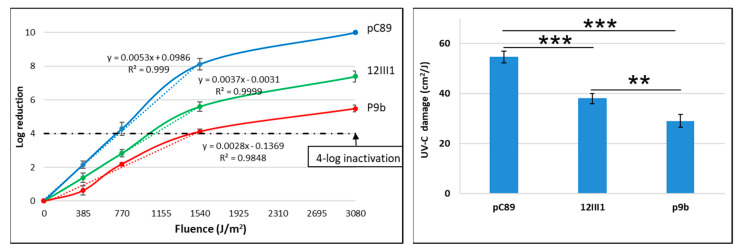
UV inactivation rates (**left** panel) and related UV-C damage (**right** panel) of pC89, 12III1 and P9b phage. The black dotted line marks the 4-log inactivation level to deduce the UV-C damage by linear trendline to the exponential phase of log-inactivation for each phage. For ANOVA test from Tukey’s multiple comparisons test, two (**) and three (***) asterisks identify *p*-value < 0.001 and 0.0001, respectively. For each point, the mean and standard deviation were derived from 5 experimental data.

UV-C inactivation rates and related UV-C damage in pC89 wild-type, P9b and 12III1 phages are reported in Figure 2.

For all phages, UV-C inactivation rate showed a logarithmic trend, consisting in a first dose-dependent exponential phase (up to 1540 J/m^2^). Specifically, the UV-C damage for each phage was determined at the threshold value of four-log reduction. pC89 showed a significantly higher value than the two engineered phages (54.5 ± 2.3 J/cm^2^) than the two engineered phages (38 ± 2 and 29 ± 2.5 J/cm^2^ for 12III1 and P9b, respectively). The difference in resistance to UV-C exposure of the phages was also maintained at 3080 J/m^2^ (maximum dose tested in our experiments). In addition, a significant difference in susceptibility to UV-C exposure was also observed between the two engineered phages (Figure 2, left panel). In fact, P9b showed the lowest UV-C damage value, that was 24% lower than 12III1 (Figure 2, right panel).

### 2.3. Hydrogen Peroxide Resistance Test

The infectivity of pC89, P9b and 12III1 phages (TU/mL) after treatment with 1.5 M H_2_O_2_ for several incubation times is reported in Table 3.

**Figure 3:**
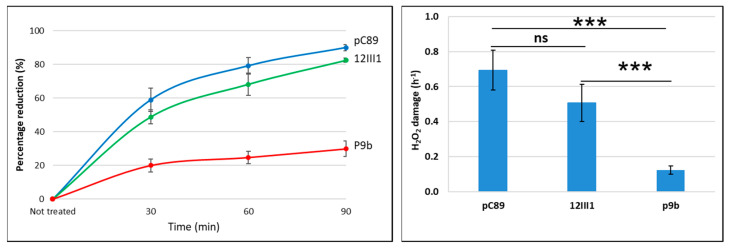
H_2_O_2_ inactivation rates (**left** panel) and related H_2_O_2_ damage (**right** panel) of pC89, 12III1 and P9b phage. For ANOVA test from Tukey’s multiple comparisons test, three (***) asterisks identify *p*-value < 0.0001. ns = not significant. For each point, the mean and standard deviation were derived from 5 experimental data.

The pC89 and 12III1 infectivity decreased by about 0.5 orders of magnitude after 30 and 60 min, and one order of magnitude after 90 min, whereas P9b was the most resistant by a significant margin, since TU maintained viability throughout all treatment durations (Figure 3).

Additionally in this case, inactivation of phages has a logarithmic trend, consisting in a first dose-dependent exponential phase in the first 30 min. In addition, P9b maintained the highest resistance to treatment, as confirmed by the lowest H_2_O_2_ damage value, that was 82% and 75% lower than pC89 and 12III1, respectively. However, respect to UV-inactivation, no significant difference between pC89 and 12III was observed, while as also confirmed by H_2_O_2_ damage (Figure 3**,** right panel).

## 3. Discussion

Some studies have reported virus inactivation after exposure to different types of radiation (i.e., electron beam, gamma irradiation, lethal ultraviolet), particularly applied to water and food safety [34] or to phage therapy to plants [35]. However, the extraordinary diversity of viruses does not allow defining universal parameters about their susceptibility to the radiation exposure. Indeed, opposing conclusions have been reported about the inactivation of bacteriophages by electron beam and gamma irradiation [29].

Since phages have limited ability to repair DNA, viruses’ resistance is closely related to the protein composition of the external structures of the virion. Although structural studies have been carried out by mutational inductions abrogating/increasing interactions within viral structures, they generally do not consider the effects of radiations on viral structures, as well as the molecular mechanisms responsible for more or less resistance to infectivity inactivation. Moreover, these studies cannot apply to engineered phages, for which the addition of amino acid sequences in viral capsid structure derive from screening/selection of engineered phages able to detect specific target, key step of research projects for the development of new diagnostic/therapeutic systems.

In this work, we investigated whether the foreign peptides in the N-terminal pVIII of the P9b and 12III1 phages, could influence the resistance to UV-C.

First of all, speculative 3D models have been used to screen the engineered phages (available in our laboratories) to employ in the biological tests. Based on the hydrogen (H) and steric bonds that could occur on the hybrid capsid structure, two engineered phage clones were chosen, because of more (P9b) and less (12III1) interactions due to their foreign amino acids. Specifically, in P9b the foreign peptide (amino acid sequence QRKLAAKLT) led to linear extension of engineered pVIIIs, respect to the pVIII wild type counterpart, with a significant increase of interactions in the hybrid capsid structure, both recombinant/wild-type and recombinant/recombinant pVIIIs (Figure 1; Table 1). Otherwise, in 12III1 the foreign peptide (amino acid sequence RWPPHFEWHFDD) induced a curvature of the engineered pVIIIs, with an increase of interaction numbers only between the recombinant pVIIIs (Figure 1; Table 1). Because of the strong interaction between capsomers and nucleic acids, the capsid rearrangement of engineered phages could protect the viral DNA from tertiary structural changes induced by the surrounding environmental stresses, such as UV-C radiation.

Results from the experimental design of radiation exposure revealed that the presence of the foreign peptide had a greater effect on phage infectivity. In fact, pC89 was almost totally inhibited after the exposure to UV-C for 1540 J/m^2^ fluence, according to the data reported previously [32]. In contrast, P9b and 12III1 phages maintained their viability under the UV-C exposure until 3080 J/m^2^ (1.4 ± 0.7 × 10^4^ and 1.7 ± 0.9 × 10^2^ TU/mL for P9b and 12III, respectively)**.** These results suggest that both the phage clones had acquired an advantage against direct radiation damage from UV-C exposure, respect to pC89. Moreover, the increase in UV-C resistance was in agreement with the increase of the interactions (predicted by the model) in the capsids of the engineered phages, as evidenced by the higher resistance of P9B compared to 12III1. H-bond and steric interactions favorably contribute to protein stability and could reduce the susceptibility of the hybrid phage structure to the modifications induced by UV-C, as also suggested by the experimental results. In fact, the number increasing of H-bond and steric interactions (pC89 < 12III1 < P9b) was positively correlated with the phage resistance to UV-C.

UV-C radiation, usually used in the disinfection of wastewater, is predominantly absorbed by nucleic acids, causing damage by the formation of photoproducts [36]. Formation of dimers (cyclobutane pyrimidine and pyrimidine) and photoproducts (pyrimidine) has been reported to increase in proportion to the UV-C dose, modifying the DNA structure. On the other hand, radiations can induce an indirect damage, due to OH^−^ and H^+^ products resulting from the water radiolysis [37]. Since the latter mainly involves protein structures, including individual amino acids, the radiolysis of water could play a role in the different behavior of the two engineered phage clones to ionizing radiation. At this purpose, the infectivity of the tested phage has also been evaluated after treatment with 1.5 M H_2_O_2_. Surprisingly, only P9b did not lose its viability, while infectivity of pC89 and 12III1 were decreased of 1 and 0.5 orders of magnitude, respectively, after 90 min of treatment (Table 3 and Figure 3). In this prospective, the amino acid composition of the foreign peptide could alter the degree of oxidative modifications in the capsid proteins, due to H_2_O_2_, which can be considered the most common indirect damage from radiation exposure. Specifically, peroxyl radicals can induce the formation of oxidation products by radical mechanisms, leading to fragmentation of the protein backbone. It is known that aromatic amino acids are significantly more reactive with the dominant reaction pathway being OH^−^ addition to the aromatic ring [38]. In this work, 12III1 phage has four aromatic amino acids in its foreign peptide, while P9b none. Consequently, hybrid phage structure of 12III1 would not have an advantage in H_2_O_2_ resistance, when compared to pC89. The remaining amino acids of the foreign peptide of 12III1 (namely arginine, proline, histidine, glutamic acid and aspartic acid) could also negatively affect the oxidation resistance of the phage clone. In fact, the radiation-generated ROS modifies protein by carbonylation to the amino-acid side chains, particularly occurring on Lys, His, Thr, Pro, Glu, Asp, and Arg residues [38]. On the other hand, amino acids sequence of the P9b foreign peptide, with only one amino acid favorable to carbonylation (namely arginine) makes the phage clone more resistant to H_2_O_2_ respect to other phage, subject of this study.

## 4. Conclusions

Phage display technique allows to screen and select peptides with several biotechnological functions. These foreign peptides are exposed in-frame inside the capsid protein pIII or pVIII. Consequently, the entire capsid structure may be affected by the onset of new interactions. In order to understand the incidence of capsid organization on resistance to environmental stresses, in this work the resistance to UV-C radiation of M13 wild-type vector (pC89) and two engineered phage clones in pVIII, expressing 9 or 12 additional amino acids in N-terminal end of pVIII capsid protein, was evaluated.

Our data indicate that the presence of the foreign peptide increase the number of interactions between the pVIII in engineered phages, bringing advantages in resistance to UV-C radiation. Moreover, among the engineered phages under study, the increase in UV-C resistance was directly linked to the increase in the number of interactions, which in turn depended on the peptide sequence of the exogenous peptide. Phages engineered to express known peptides on their capsid could represent models extremely useful to understand the molecular mechanisms involved in the stability of new virus variants, also providing quick and necessary information to develop effective protocols in the virus inactivation for human activities, such as safety foods and animal-derived materials.

## 5. Materials and Methods

### 5.1. Bacteriophages

Phages used in this work were obtained from two M13 phage display libraries (kind gift of Prof. F. Felici). These libraries consist of filamentous phage particles displaying random 9- or 12-mer peptides fused to the major coat protein (pVIII). The libraries were constructed in the vector pC89 [39], by cloning a random DNA insert between the third and fifth codon of the mature pVIII-encoding segments of gene VIII [40]. pC89 (M13 wild-type vector without peptide insert) and two engineered phage clones with different amino acid sequences of the foreign peptide were used. Specifically, the P9b phage clone displayed the sequence QRKLAAKLT [41], while the 12III1 the sequence RWPPHFEWHFDD [42]. Propagation of pC89 and engineered phage clones was carried out in *Escherichia coli* TG1 bacterial host (Lac-Z deleted).

### 5.2. Phages Production

*E. coli* strain TG1 (Kan-, Amp-, lacZ-) broth culture (OD_600_ = 0.7) was infected with pC89 or engineered phage clone (Amp+), then incubated at 37 °C in static condition for 15 min, followed by shaking (250 rpm) for 20 min. After incubation, suitable aliquots of culture were plated onto Luria–Bertani agar (agar 20 g/L) plates containing ampicillin (50 µg/mL) and incubated at 37 °C in static condition. One colony of *E. coli* strain TG1, containing phage, was inoculated into 10 mL of LB medium containing ampicillin (50 µg/mL) and incubated at 37 °C with shaking (250 rpm) until reaching OD_600_ = 0.2. Then, the culture was added with isopropylthio-β-galactoside (IPTG, 40 µg/mL) and helper phage M13K07 (Kan+) (10^9^ TU/mL), incubated at 37 °C in static condition for 30 min, and gently shaken for 30 min. The cells were harvested by centrifugation at 8000× *g*, transferred to 500 mL of LB medium containing ampicillin (50 µg/mL) and kanamycin (10 µg/mL), and incubated overnight with shaking at 37 °C. The infected culture was centrifuged 8000× *g* for 20 min at 25 °C, the supernatant was then mixed with 25% (*v*/*v*) of PEG/NaCl solution, cooled in ice for 4 h, and precipitated by centrifugation at 15,000× *g* for 45 min at 4 °C. The pellet was resuspended in 10% (*v*/*v*) of TBS, mixed again with 25% (*v*/*v*) of PEG/NaCl, cooled in ice for 4 h, and the solution was centrifuged as above. The pellet, containing phage particles, was suspended in 10% (*v*/*v*) of TBS, filtered through 0.22 µm-pore size membrane, and stored at 4 °C.

### 5.3. In Silico 3D Modelling Analysis

Engineered pVIII Protein Structure Modeling was performed using the MODELLER9.20 (https://salilab.org/modeller, (accessed on 15 March 2021).) software for the comparative protein structure modeling. The models were built as reported previously [43]. Briefly, the structure of pVIII protein (PDB ID: 2mjz) was obtained. In the context of the whole virus particle of 2mjz, two pVIII proteins, Chain [1a] and Chain [1e], have been chosen for modeling the pVIII engineered proteins. Since these proteins are located at the same position between pentamer rings 1° and 3°, they allow identification of the contributing protein-protein interactions in the capsid structure [6]. The two pVIII proteins were kept like the single pVIII protein, in a PDB-format, namely chain A and chain E, and used as templates. Amino acid sequences of engineered pVIII proteins of P9b and 12III1 clones, with a foreign peptide inside of fourth/fifth amino acids of the M13 wild-type pVIII (plus two extra residues of Phenylalanine and Glutamine, encoded by the EcoRI site as reported in Felici et al. [39] were written on the following FASTA format to derive pVIII-engineered models (in bold the foreign amino acid sequence):

1-AEGDDPAKAAFNSLQASATEYIGYAWAMVVVIVGATIGIKLFKKFTSKAS-50 (pC89);
1-AEGEFQRKLAAKLTDPAKAAFNSLQASATEYIGYAWAMVVVIVGATIGIKLFKKFTSKAS-60 (P9b);
1-AEGEFRWPPHFEWHFDDGDPAKAAFNSLQASATEYIGYAWAMVVVIVGATIGIKLFKKFTSKAS-64 (12III1).

Several models were calculated for the same target. The best 3D model was selected, according the lowest value of DOPE, which indicates the construction energy. For each engineered pVIII protein, two models were obtained, one from the chain A and one from the chain E of the capsid complex.

### 5.4. Analysis of Amino Acids Involved in the Structure Capsid Interactions

The models performed in PDB format were opened and processed using Molegro Molecular Viewer v1.2.0 (http://www.molegro.com (accessed on 20 February 2020)). The models were processed as ligand-protein in “Energy Map” and “Ligand Map” to identify the amino acids involved in the interactions. ProtParam (https://web.expasy.org/protparam (accessed on 20 February 2020)) bioinformatics tool was used to predict the following characteristics: molecular weight, theoretical pI, amino acid composition, atomic composition, extinction coefficient, estimated half-life, instability index, aliphatic index, and grand average of hydropathicity (GRAVY) of the amino-acid sequence of the peptide [44].

### 5.5. Virus Irradiation

Due to the extremely low penetrative ability of the UV-C radiation, were separately placed in uncovered petri dishes (sample exposure surface and thickness approximately 0.8 cm^2^ and 0.15 cm, respectively) and exposed to UV-C radiation (G15T8/OF, OSRAM germicidal, puritec HNS 15WG13, operating with the emission line at the 253.7 nm wavelength) for different time exposure at a distance of 150 mm. Specifically, the germicidal lamp has a UV output power of 4.9 W and an intensity of 50 µW/cm^2^ at a 1-m distance. Since the intensity decreases with the square of the distance between the lamp and the target, at a 15 cm distance from our operation, the lamp intensity is 2.2 mW/cm^2^. All irradiations were carried out in the biological hood at 25 °C. After the treatment, the infective ability of recovered phages was evaluated by titration. All tests were performed in quintuplicate.

### 5.6. Hydrogen Peroxide Resistance Assay

Hydrogen peroxide (H_2_O_2_) was evaluated at a sub-lethal dose, according to Eisenstark et al. [45]. In detail, 30% stock solution of H_2_O_2_ was diluted with sterile double distilled water immediately prior to each experiment. Each phage stock (1 × 10^11^ phage/mL) in TBS (100 µL) was treated with 1.5 M H_2_O_2_ (final concentration) for 30, 60, and 90 min. At each time, 10 µL catalase (2 mg/mL) were added to stop the reaction. All tests were carried out in the biological hood at room temperature. After the treatments, the infective ability of the recovered bacteriophages was evaluated by titration, as described below. All tests were performed in quintuplicate.

### 5.7. Phage Titration (TU/mL)

Tenfold serial dilutions of pC89 or engineered phage clones were prepared, and 10 µL of each phage sample were dispensed into sterile micro-centrifuge tubes containing 90 µL of *E. coli* TG1 culture (OD_600_ = 0.7). Tubes were first incubated at 37 °C for 15 min in static conditions and then for 20 min in shaking (250 rpm) conditions. 100 µL of pC89/*E. coli* TG1 or engineered phage/*E. coli* TG1 suspension was spotted onto LB agar plates containing ampicillin (50 µg/mL). All plates were incubated at 37 °C overnight. Colonies from plates (between 30–300) were counted and the number of active virus particles was determined as Transducing Unit per milliliter (TU/mL) according to the following Equation (1)

(1) $$\mathrm{TU}=\frac{\mathrm{number}\;\mathrm{of}\;\mathrm{colonies}}{\mathrm{volume}\;\left(0.1\;\mathrm{mL}\right)\times \mathrm{diluition}\;\mathrm{factor}}$$

All assays were performed in quintuplicate.

Using the TU/mL value, the damage from UV-C or H_2_O_2_ per fluence (J/m^2^) or time (h) was derived from the following Equation (2)

(2) $${\mathrm{UVC}\;\mathrm{or}\;H}_{2}O_{2}\;\mathrm{damage}=\frac{\log_{10}\mathrm{Ns}\left(r\right)-\log_{10}\mathrm{Ns}\left(s\right)}{\mathrm{fluence}\;\left(\frac{J}{{\mathrm{cm}}^{2}}\right)\;\mathrm{or}\;\mathrm{time}\;\left(h\right)}$$

where Ns(r) and Ns(s) represent the number of active virus particles (TU/mL) not irradiated and irradiated, respectively. The significant differences in the UVC or H_2_O_2_ damage values were determined by analysis of variance (ANOVA) using adjusted *p*-values at 0.01 and 99% for family-wise significance and confidence level, respectively.

## Figures and Tables

**Table 1:** Position number in the primary sequence of amino acids involved in the interactions between PVIII proteins in pC89, P9b and 12III1 phages, deduced by in silico 3D modeling analysis (Figure S2).

	pC89	P9b (QRKLAAKLT)		12III1 (RWPPHFEWHFDD)	
	Wild-Type/ Wild-Type pVIIIs	Recombinant/ Wild-Type pVIIIs	Recombinant/ Recombinant pVIIIs	Recombinant/ Wild-Type pVIIIs	Recombinant/ Recombinant pVIIIs
H-bond	43	42–43, 45–46, 49	1–2, 4–5, 7–10, 22–23, 31, 42, 45–46, 53	14	12–13, 40–42, 45–47, 49–54
Steric interactions	21, 28, 32, 35–36, 39	1, 4–5, 7–8, 26–27, 30–31, 34, 37–46, 48–50, 52–53	1–12, 14–16, 18–20, 22–24, 26–28, 30–32, 34, 38, 41–43, 45–50, 53	13–17, 20, 31	9–14, 34, 38, 40–54

**Table 2:** Active virus particles of pC89, P9b and 12III1, expressed as Transducing Unit per milliliter of infected *E. coli* (TU/mL), after exposure to UV-C.

Phage	Exposed	385 J/m^2^	770 J/m^2^	1540 J/m^2^	3080 J/m^2^
pC89	(4 ± 0.9) × 10^9^	(3 ± 1.7) × 10^7^	(2.2 ± 1.2) × 10^5^	(3.1 ± 1.3) × 10	No TU
P9b (QRKLAAKLT)	(3.9 ± 0.2) × 10^9^	(1.1 ± 0.6) × 10^9^	(2.6 ± 0.7) × 10^7^	(3.2 ± 1.3) × 10^5^	(1.4 ± 0.7) × 10^4^
12III1 (RWPPHFEWHFDD)	(3.9 ± 0.9) × 10^9^	(1.8 ± 0.9) × 10^8^	(5.7 ± 1.4) × 10^6^	(1.2 ± 1) × 10^4^	(1.7 ± 0.9) × 10^2^

**Table 3:** Active virus particles of pC89, P9b and 12III1, expressed as Transducing Unit per milliliter of infected *E. coli* (TU/mL), after exposure to H_2_O_2_ (1.5 M).

Phage	Exposed	30 Min	60 Min	90 Min
pC89	(1.2 ± 0.2) × 10^10^	(5 ± 0.4) × 10^9^	(2.5 ± 0.3) × 10^9^	(1.2 ± 0.2) × 10^9^
P9b (QRKLAAKLT)	(4.0 ± 0.3) × 10^10^	(3.2 ± 0.4) × 10^10^	(3 ± 0.3) × 10^10^	(2.8 ± 0.1) × 10^10^
12III1 (RWPPHFEWHFDD)	(3.4 ± 0.2) × 10^10^	(1.7 ± 0.2) × 10^9^	(1.1 ± 0.2) × 10^9^	(5.7 ± 0.6) × 10^9^

## Supplementary Materials

The following are available online at https://www.mdpi.com/article/10.3390/ijms22073408/s1, Figure S1: 3D models of the pVIII protein chain A in pC89 (A), P9b (B) and 12III1 (C), with the amino-acids of the foreign peptide in wireframe style. (PDB ID: 2mjz, Morag et al., 2015). Figure S2: Ligand map from 3D models of the interactions in pC89, P9b and 12III1 phage, including abbreviation and position of the amino acids involved in H-bond and steric interactions.
