# Supplementary material for: Role of Phage Capsid in the Resistance to UV-C Radiations
Source: Int J Mol Sci. 2021 Mar 26;22(7):3408. doi: 10.3390/ijms22073408 (PMC8037334; doi:10.3390/ijms22073408)
Supplement: Supplementary file 1 [file ijms-22-03408-s001.zip › ijms-1148462-supplementary/Revised ijms-1148462_SM.docx]

Supplemental material

Role of phage capsid in the resistance to UV-C radiations

**Laura Maria De Plano^1^, Domenico Franco^1^*, Maria Giovanna Rizzo^1^, Vincenzo Zammuto^1^, Concetta Gugliandolo^1^, Letteria Silipigni^2^, Lorenzo Torrisi^2^ and Salvatore P.P. Guglielmino^1^**

^1^ Department of Chemical, Biological, Pharmaceutical and Environmental Sciences, University of Messina, Italy; [ldeplano@unime.it](mailto:ldeplano@unime.it) (L.M.D.P.); [dfranco@unime.it](mailto:dfranco@unime.it) (D.F.); [mgrizzo@unime.it](mailto:mgrizzo@unime.it) (M.G.R.); [vzammuto@unime.it](mailto:vzammuto@unime.it) (V.Z.); [cgugliandolo@unime.it](mailto:cgugliandolo@unime.it) (C.G.); [sguglielm@unime.it](mailto:sguglielm@unime.it) (S.P.P.G.)

^2^ Department of Mathematical and Computational Sciences, Physical Sciences and Earth Sciences, University of Messina, Italy; [lsilipigni@unime.it](mailto:lsilipigni@unime.it) (L.S.); [ltorrisi@unime.it](mailto:ltorrisi@unime.it) (L.T.)

* Correspondence: [dfranco@unime.it](mailto:dfranco@unime.it)

**Results**


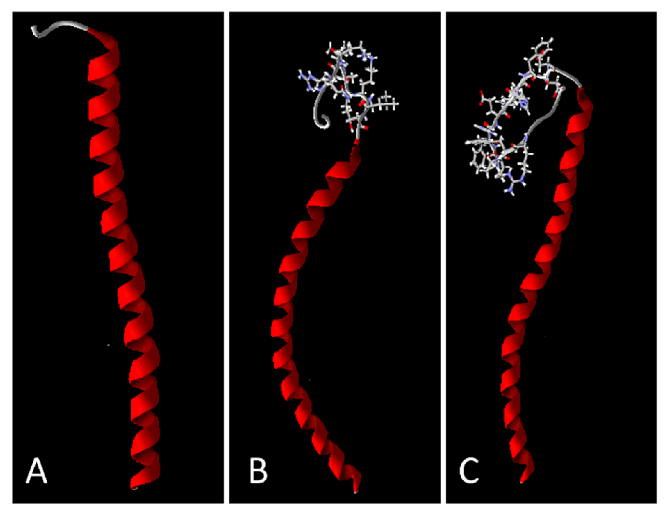


**Figure S1** 3D models of the pVIII protein chain A in pC89 (A), P9b (B) and 12III1 (C), with the amino-acids of the foreign peptide in wireframe style. (PDB ID: 2mjz, Morag *et al*., 2015).


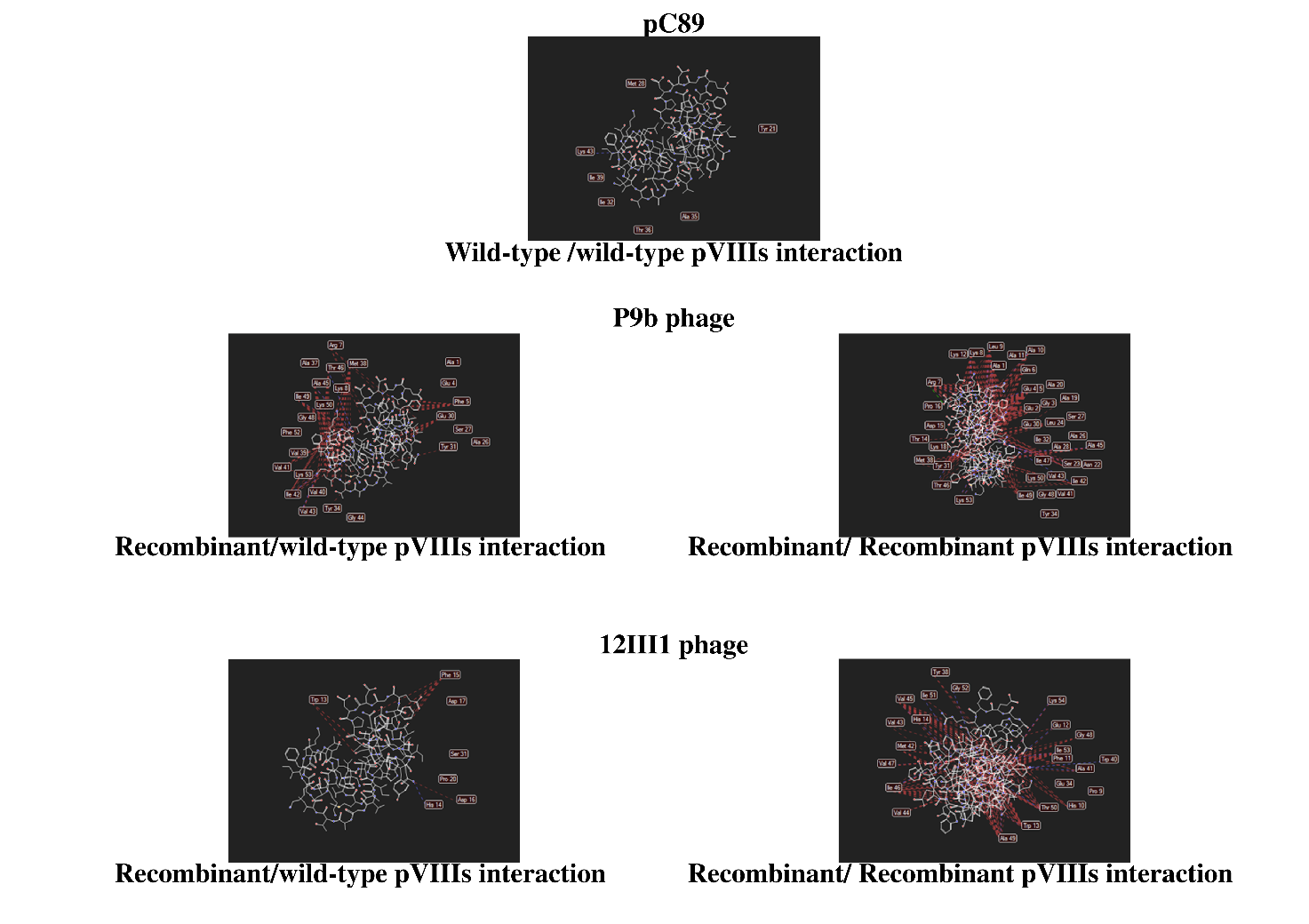


**Figure S2** Ligand map from 3D models of the interactions in pC89, P9b and 12III1 phage, including abbreviation and position of the amino acids involved in H-bond and steric interactions.
