# Supplementary figures and images for: Role of Phage Capsid in the Resistance to UV-C Radiations
Source: Int J Mol Sci. 2021 Mar 26;22(7):3408. doi: 10.3390/ijms22073408 (PMC8037334; doi:10.3390/ijms22073408)

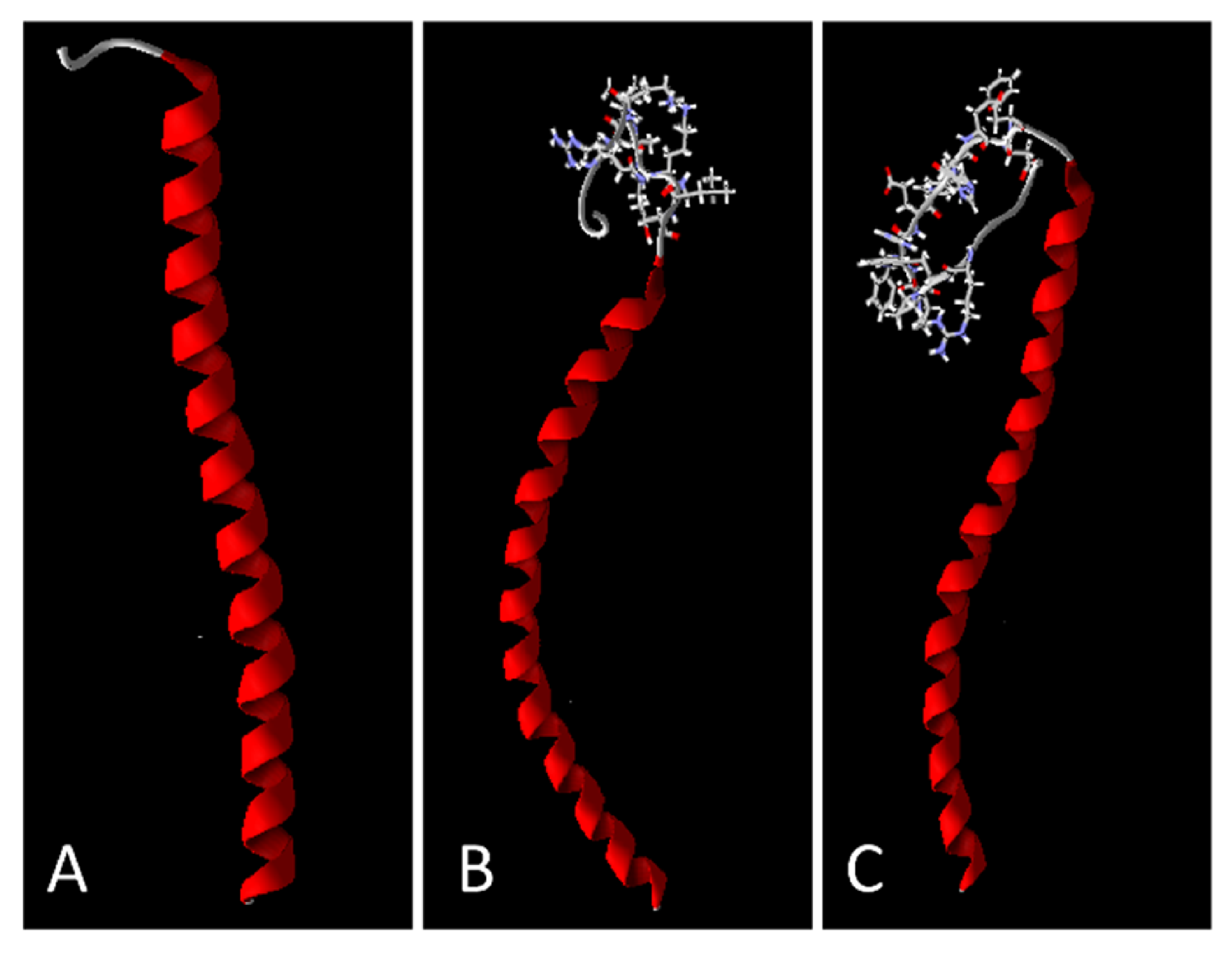

Supplement: Supplementary file 1 [file ijms-22-03408-s001.zip › ijms-1148462-supplementary/Figure S1.tif]

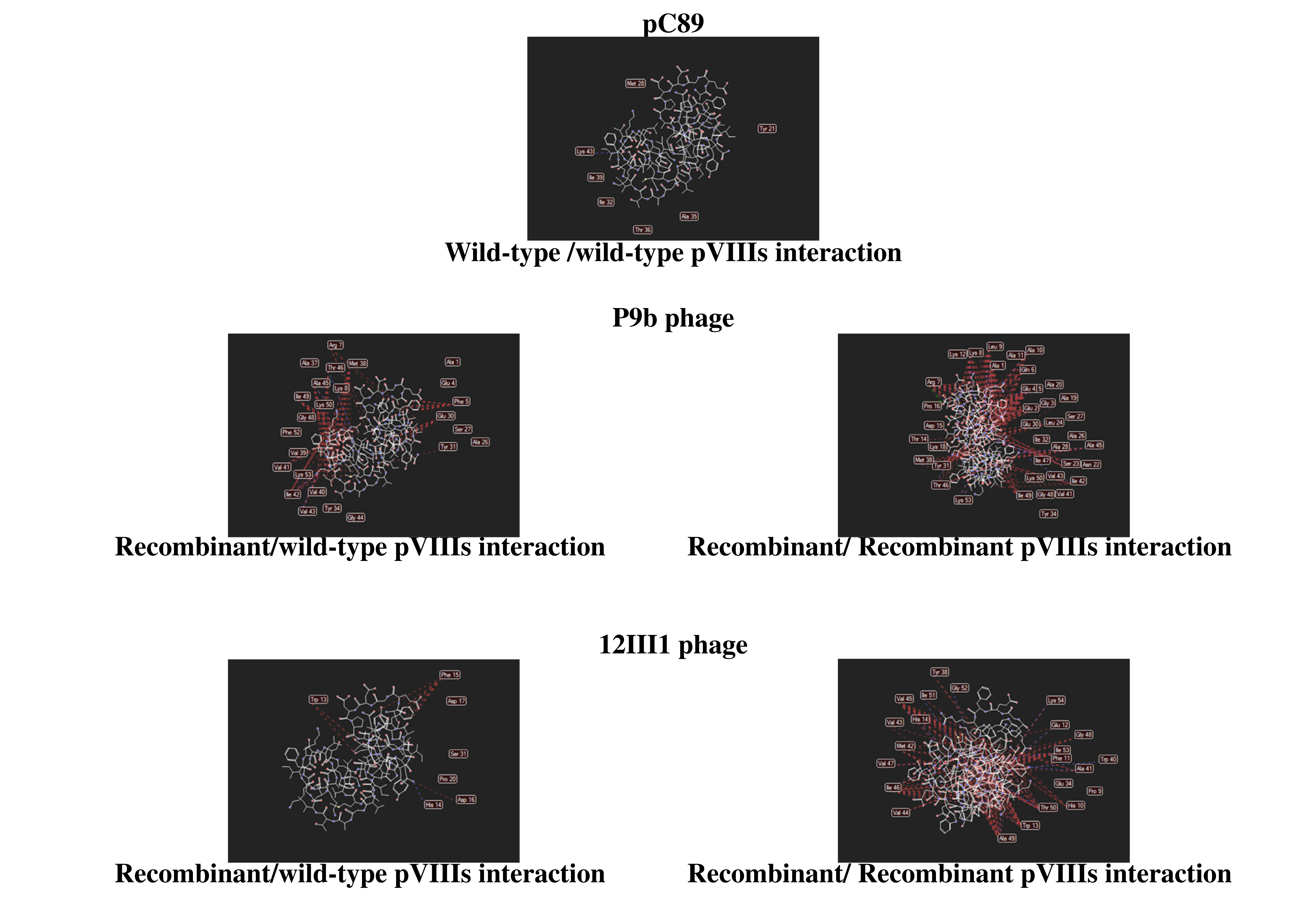

Supplement: Supplementary file 1 [file ijms-22-03408-s001.zip › ijms-1148462-supplementary/Figure S2.tif]
